# Psychological Anxiety of College Students' Foreign Language Learning in Online Course

**DOI:** 10.3389/fpsyg.2021.598992

**Published:** 2021-05-28

**Authors:** Xue Wang, Wei Zhang

**Affiliations:** ^1^Department of Western Language, Mudanjiang Normal University, Mudanjiang, China; ^2^Department of Computer Science and Information Technology, Mudanjiang Normal University, Mudanjiang, China

**Keywords:** online course, foreign language learning, anxiety, psychology, data mining, effective ways

## Abstract

Anxiety is one of the most important affective factors affecting college students' foreign language learning. Especially in the Internet age, new teaching ideas and methods bring new load and anxiety to students' psychology. Taking students who attend a college English online course learning as the research object, this paper analyzes the general situation and professional skills of the students' psychological anxiety under the network environment by using the method of investigation and data analysis. It conceives six methods to reduce the students' psychological anxiety according to the reason analysis and summarizes the more effective ways with data mining of another questionnaire. It points out that teachers can advocate the mode of group learning and peer cooperation, strengthen the timeliness and diversity of tests, increase the richness of extracurricular activities, and increase teachers' and students' quality of online teaching and learning to reduce the anxiety of students' foreign language learning in an online teaching environment.

## Introduction

Nowadays, the network has gradually penetrated into all aspects of people's life, and education is no exception. Online teaching mainly serves as the beneficial supplement of offline courses or one form of blended teaching before, but the sudden outbreak of the epidemic makes online teaching go to the stage completely. During this period of national online course, the online course brings not only infinite convenience but also certain changes to the students' psychology in the long-term online learning process. In particular, foreign language teaching has its own unique discipline nature, which requires a high level of interaction between teacher and students and among students. However, due to the lack of a cordial and effective interaction in actual communication, many students will have additional psychological burden, which will cause psychological anxiety for a long time.

### Anxiety

According to Freud (1936), anxiety is a kind of complex emotional experience that can be described by “tension, uneasiness, worry, worry,” which has a certain hint of a dangerous situation. From the perspective of psychology, anxiety is a kind of stress instinct of the human body. Moderate anxiety is “psychological immunization,” but long-term anxiety will lead to irritability, persistent fear, pessimism and depression, and the like. From the perspective of clinical medicine, excessive anxiety will bring a series of hazards to the individual's body, psychology, and behavior. When the body is in a long-term state of anxiety, it will lead to individual panic, blood pressure rise, dizziness, blurred vision, tinnitus, and so on. This paper points out that anxiety is a kind of psychology that will do great harm to the human body and cause changes in people's behavior. Long-term anxiety will make individuals lose confidence and make it difficult for them to face setbacks and pressure. Anxiety was firstly considered a concern by people in the 1840's, and it is the emotional reflection of people's serious deterioration of the value characteristics about reality or future things. It is also the complex emotional state of tension, uneasiness, anxiety, worry, and other unpleasant feelings caused by an upcoming danger or threat. In the 1950–1960's, research on the relationship between anxiety and learning outcomes began to emerge (Spielberger, 1966) and gradually increased in the 1970's.

### Foreign Language Learning Anxiety

In 1986, Horwitz, an American psychologist, first proposed the concept of “foreign language classroom anxiety.” He believed that foreign language classroom anxiety is “a significant self-perception, belief and emotion related to classroom learning and generated in the process of learning this language.” It is likely to be a ubiquitous emotional factor, which hinders the development of learning (Arnold and Brown, 1999). It was found that students with foreign language learning anxiety are prone to produce negative emotions such as tension, worry about foreign language, fear, and so on in a foreign language teaching environment, especially in the process of English practice. They often show symptoms such as not speaking, much tenseness, uneasiness, fear and heart rate acceleration, sweating, and so on, which are important factors that affect students' learning. It has been found that Language anxiety is a central factor that influences the abilities of foreign language learners in all areas (Argaman and Abu-Rabia, 2002). For this reason, anxiety has become one of the focuses in second language acquisition research.

Horwitz et al. (1986) divides English classroom anxiety into three dimensions according to its performance: communicative anxiety, negative evaluation anxiety, and test anxiety. Communicative anxiety refers to the degree of fear or anxiety about real or expected communication with others. According to the research of MC Lipsey et al. (1985), the typical behavior pattern of communication phobia is communication avoidance or withdrawal. Compared with those who have no fear, those who are afraid of communication are more reluctant to intervene in other people's conversation and pursue social interaction. Fear of negative evaluation refers to the feeling of fear of others' evaluation, depression of negative evaluation, and fear that others will make a negative evaluation of themselves. Test anxiety refers to students' tendency to view insufficient results in the process of investigation with fear (Fraser, 1981). In other words, students are worried about the exam. Culler and Holahan (1980) speculates that test anxiety may be caused by students' lack of language ability or by students' over-recollection of their failure experience. Macintyre and Gardner (2010) classified anxiety into trait anxiety, state anxiety, and specific situation anxiety according to different situations. Trait anxiety refers to the long-term anxiety inherent in people's character (Young, 1986). Trait anxiety occurs in all situations. There is a strong correlation between state anxiety and trait anxiety. Specific situation anxiety is a kind of anxiety that individuals produce in a specific situation for a long period of time. It is caused by a special event or a specific situation (MacIntyre and Gardner, 1994). Sometimes this kind of emotion can also be considered as state anxiety in a specific situation. Specific situation anxiety is different from the first two kinds of anxiety, which mainly emphasizes the relatively independent anxiety stimulation situation. Some scholars also divided anxiety into facilitative anxiety and degenerative anxiety. The former motivates learners to overcome the difficulties encountered in the process of language acquisition, challenges new learning tasks, and thus overcomes anxiety; the latter leads learners to escape anxiety by avoiding learning tasks (Spielberger and Gorsuch, 1983). However, a large number of studies have proved that anxiety has no promoting effect in English learning, and there is a negative correlation between anxiety and foreign language learning, that is to say, anxiety will have a negative impact on foreign language learning, which is supported by this paper too.

In 1991, Horwitz and Yong edited the book *Foreign Language Anxiety: From Theory and Research to Classic Influence*, marking that the study of foreign language anxiety has entered a relatively mature period. There has been no interruption in the study of foreign language learning anxiety. Scholars at home and abroad have conducted a lot of research and measurements on foreign language learning anxiety from different perspectives, such as the study of foreign language learning anxiety from the five basic skills of foreign language and the study of foreign language learning anxiety categories in blocks (Nia et al., 2019). Among them, the FLCAS compiled by Horwitz et al. up to now, is a relatively perfect special scale that has been widely used in the study of foreign language learning anxiety. Scholars have done a lot of research and measurement on foreign language learning anxiety from different perspectives and found that foreign language learning anxiety has an inevitable relationship with the overall effect of foreign language learning (Coulombe, 2001; Liu and Jackson, 2008; Yan and Horwitz, 2008; Marcos-Llinás and Garau, 2009; Roghayeh and Farvardin, 2017; Teimouri, 2018; Botes et al., 2020) and the cultivation of specific language skills in foreign language learning, such as listening skills (Elkhafaifi, 2005; Bekleyen, 2009), oral skills (Sellers, 2000; Jiang and Dewaele, 2020), reading skills (Zhao, 2009), and writing skills (Cheng et al., 2019), and also affect students' self-esteem (Horwitz et al., 1986) and self-confidence (MacIntyre and Gardner, 1991). Although some studies have shown that moderate anxiety contributes to foreign language learning (Park and French, 2013), most studies have confirmed that the impact of learning anxiety on foreign language learning is negative. There is a negative correlation between learning anxiety and students' academic performance, examination performance, oral and written expression ability, and self-confidence (Gardner and MacIntyre, 1993). Besides some research has attempted to discuss sources of language learning anxiety (Mak and White, 1997).

### Students' Psychological Anxiety Toward Online Foreign Language Course

Anxiety is closely related to environment (Gardner and MacIntyre, 1993: 284). With the rapid development of information technology and the wide application of online courses, the network multimedia environment is very different from the traditional teaching environment, which is bound to have an impact on learners' emotions. Internet learning anxiety is considered as “environmental anxiety” in the process of information processing, which is caused by various uncertain and fuzzy factors in the Internet learning environment. We think that Internet learning anxiety is an emotional response of learners in the learning process under the network environment, which is caused by information overload, various uncertain factors in network learning, and difficulties in the learning process. It is a kind of anxiety phenomenon under specific environmental conditions. In fact, in the information age, network course has appeared in people's vision as an important way of learning (Horwitz, 1995). However, because it has not been the mainstream teaching method and has rarely been the main body of school teaching, the anxiety of students in online learning is not obvious, and people pay little attention to it. There is also very little research. However, in the epidemic situation today, online courses have become the only way for students to learn, so the psychological changes brought by online courses have become prominent. Especially the long-term online teaching brings students' psychological anxiety, which has become a problem that cannot be ignored (Miyazoe and Anderson, 2011).

## Materials and Methods

### Participants

The subjects of this study are four classes in a university in H Province of China. There are 40 students in each natural class. They are between 19 and 22 years old, in the basic standard age range and with no older or younger ones. There are 72 boys and 88 girls totally; one class was for liberal arts and one class was for science from freshmen, and one class was for liberal arts and one class was for science from sophomore, respectively. In the first survey, 23 invalid questionnaires were removed, and 137 valid questionnaires were obtained; in the second survey, 160 valid questionnaires were obtained, and 15 different sets of countermeasures were found.

### Materials

#### Questionnaire

Two questionnaires were conducted in this paper. Firstly, based on Horwitz's FLCAS, this paper designed a questionnaire that integrated elements of foreign language teaching and online course. Besides this, due to the important role that online learning anxiety plays in cultivating and developing students' specific foreign language skills, this paper integrates five basic skills in detail to be able to identify students' anxiety on each learning content such as listening, speaking, reading, writing, and translating more clearly and accurately. Therefore, the questionnaire was designed from the following five dimensions: online learning anxiety, communicative anxiety, examination anxiety, negative evaluation anxiety, and online skill anxiety for one hand; in addition, it can be analyzed from the perspective of five language skills on the other hand. There are 33 questions in total, with five to nine questions in each dimension, and five options are reserved for each question, which are “totally agree, agree, uncertainly, disagree or totally disagree.” The scores are five points, four points, three points, two points, and one point. According to the anxiety level, one point is lowest anxiety, and five points is highest anxiety. The average anxiety is an indicator to measure the anxiety level of each question. With reference to Horwitz and other professional literature, 3 is considered as the boundary between low and moderate anxiety, and three points belong to low-level anxiety. The total score of 33 questions is between 33 and 165 points. The higher the score is, the higher a student's anxiety is. The reliability and validity of the questionnaire are tested to test the rationality of the questionnaire (the reliability and validity are all above 0.75). Secondly, the paper conducts a questionnaire survey on the six strategies summarized through cause analysis, interview, and teaching practice before exploring the optimal set of effective ways for students' foreign language learning anxiety in the network environment. Students can choose one or more countermeasures. After the questionnaire has been collected, different sets of effective methods will be formed, and repeated combinations will be eliminated.

#### Interview

This part adopts semi-structured interview methods, and the interview adopts open proposition, focusing on the causes of students' online foreign language learning anxiety and the corresponding countermeasures. The interviewees are 80 students (20 students in each class) and 30 teachers who teach college English in two grades. Both teachers and students put forward their own ideas and suggestions on the causes and solutions of students' online foreign language learning anxiety. Each interview is for 5–10 min.

#### Procedure

After the completion of the questionnaire design, a small-scale test was conducted to test the scientificity and rationality of the questionnaire. The results show that both the reliability and validity of the questionnaire meet the requirements (all above 0.75). Then, the questionnaire was distributed in a large area. The subjects were asked to read the instruction carefully firstly and then complete the whole questionnaire according to their actual situation. The questionnaire is completed in groups with the class as the unit, and basic information, such as grade and gender, is required to be filled in. It takes about 20–30 min to complete. Finally, spss20.0 and Excel were used to analyze the data. The interview was completed within 1 month after the questionnaire survey. The results of the interview were included in the further analysis and combined with the results of the questionnaire to form the causes and countermeasures. The effective ways to deal with online foreign language learning anxiety are distributed again in the form of a questionnaire, and different sets of countermeasures are obtained. The optimal set of effective ways is calculated by using the algorithm of data mining, which will be the main method to solve the foreign language classroom anxiety in the network environment.

## Results

Considering that some scholars believe that gender factors will have an impact on students' foreign language learning anxiety (Dewaele & Macintyre) and the subjects include two liberal arts and science classes, this paper makes a regression analysis based on the proportion of male college foreign language learners and the proportion of liberal arts foreign language learners. The results of the samples show that gender and discipline are not the factors influencing the level of foreign language learning anxiety (*P* > 0.05) in this study, which is consistent with the research of Botes et al. (2020) and Tsai (2018). Therefore, this paper does not consider the gender and discipline differences in the analysis process, which means to analyze students' foreign language learning anxiety in online courses as a whole.

### Types of Students' Foreign Language Learning Anxiety

**Figure 1:**
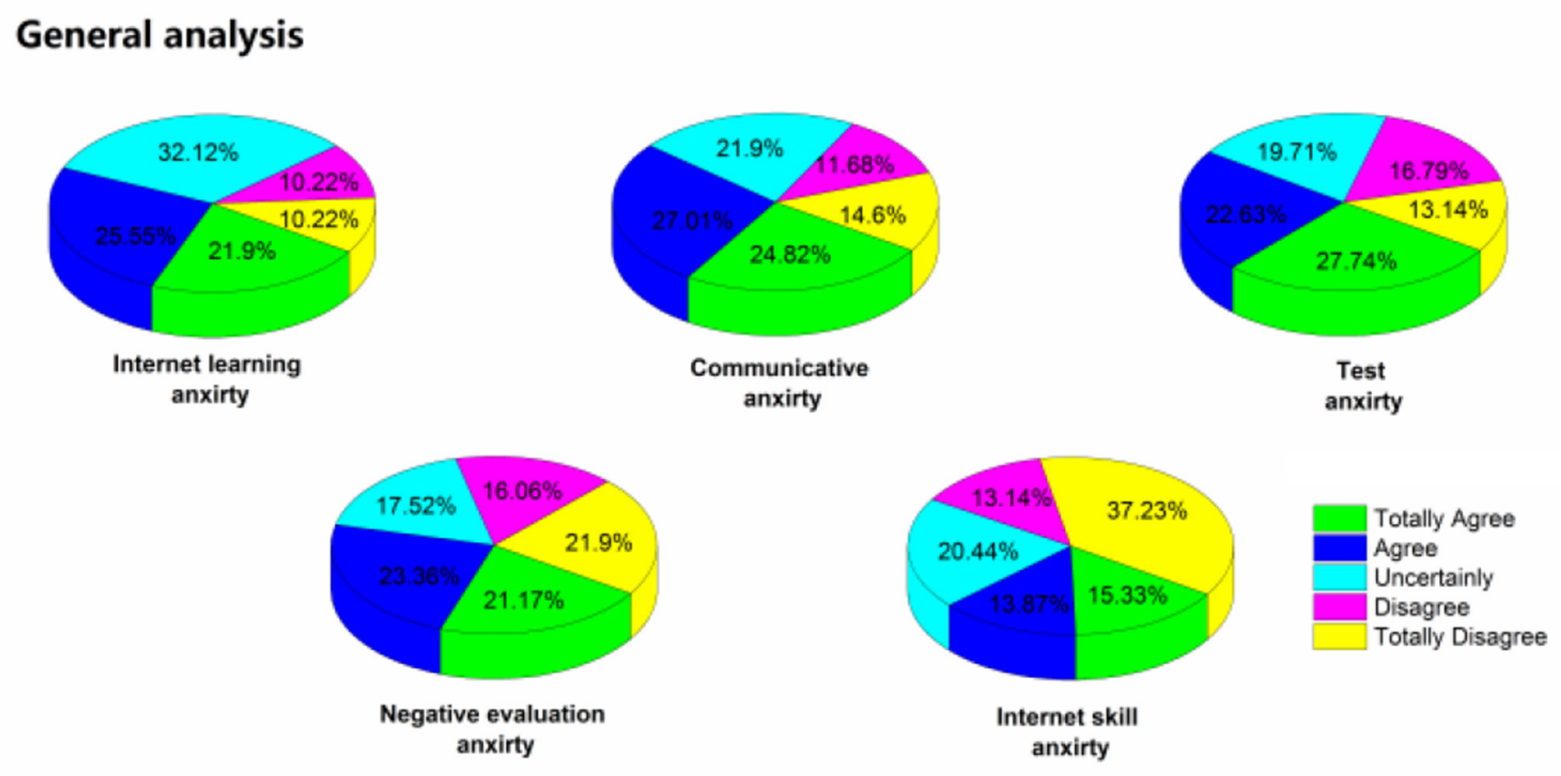
Proportion of different types of anxiety.

Through statistical analysis and calculation, the results are shown in Table 1, the mean value of anxiety is 3.145, which is higher than the middle level. We divided 33 questions into five dimensions for quantitative analysis. E-learning anxiety is mainly about the overall learning environment, learning atmosphere, and learning style, with an average of 3.387, which is the highest of the five dimensions. At present, students are quite used to traditional classroom teaching, and they are mainly worried about the form of e-learning and how to integrate it quickly. Communicative fear is mainly the anxiety of online communication and oral expression and discussion, with an average value of 3.358, which is also a relatively high anxiety value. Communication is indeed a difficult problem to solve in online courses, and it is difficult to achieve real-time communication as that in the physical classroom. Test anxiety is mainly about test form, test content, and test fairness, with an average of 3.350. The fear of negative evaluation is mainly the anxiety of evaluation form and evaluation for curriculum performance, with an average of 3.058. Network skill anxiety mainly refers to the anxiety of operation problems in network environment, network equipment, and network learning. The average value is 2.569, which is relatively low. It is mainly because the use of computer equipment and network by college students is relatively common at present, and they have mastered the ability of information processing, so the anxiety value is relatively low. The Figure 1, 2 show the specific proportion of different types of anxiety and their comparison.

**Table 1:** Distribution data of different anxiety types.

***N***	**Type**	**Totally agree**	**Agree**	**Uncertain**	**Disagree**	**Totally disagree**	**Average**
		**(%)**	**(%)**	**(%)**	**(%)**	**(%)**	
1	Internet learning anxiety	21.90	25.55	32.12	10.22	10.22	3.387
2	Communicative anxiety	24.82	27.01	21.90	11.68	14.60	3.358
3	Test anxiety	27.74	22.63	19.71	16.79	13.14	3.350
4	Negative evaluation anxiety	21.17	23.36	17.52	16.06	21.90	3.058
5	Internet skill anxiety	15.33	13.87	20.44	13.14	37.23	2.569

**Figure 2:**
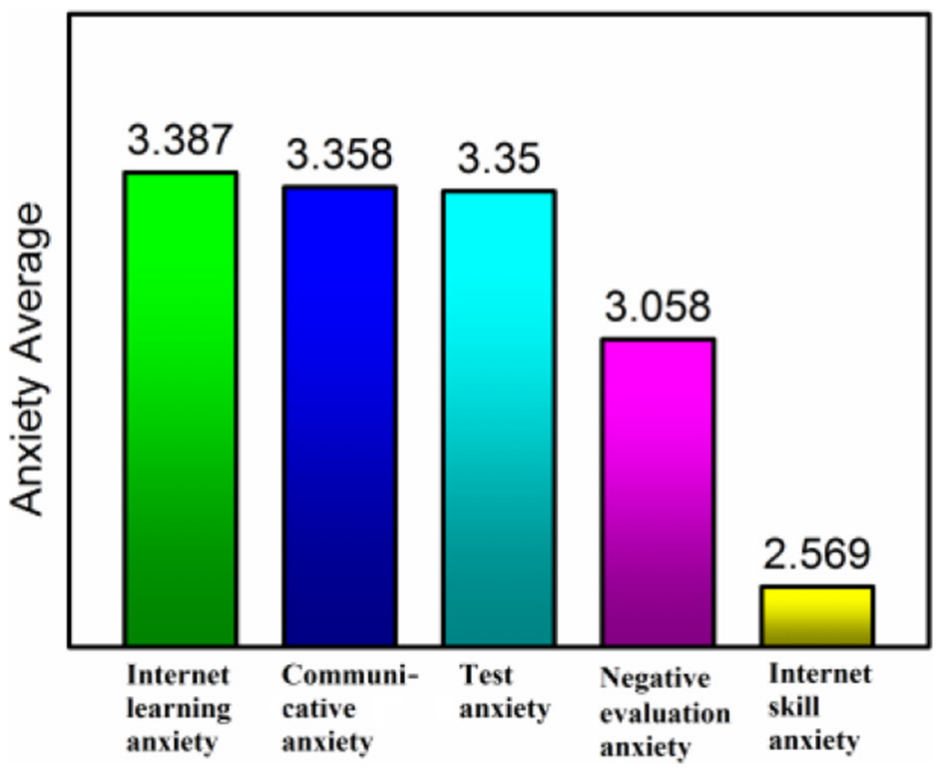
Comparison of different types of anxiety.

### Anxiety of Specific Language Skills

Foreign language teaching involves listening, speaking, reading, writing, and translating. According to the content of foreign language teaching design, 33 problems are reclassified. According to the five dimensions of listening, speaking, reading, writing, and translation, students' anxiety about professional skills involved in teaching is obvious, which is more conducive to finding solutions in this regard (Wilson et al., 2020).

**Figure 3:**
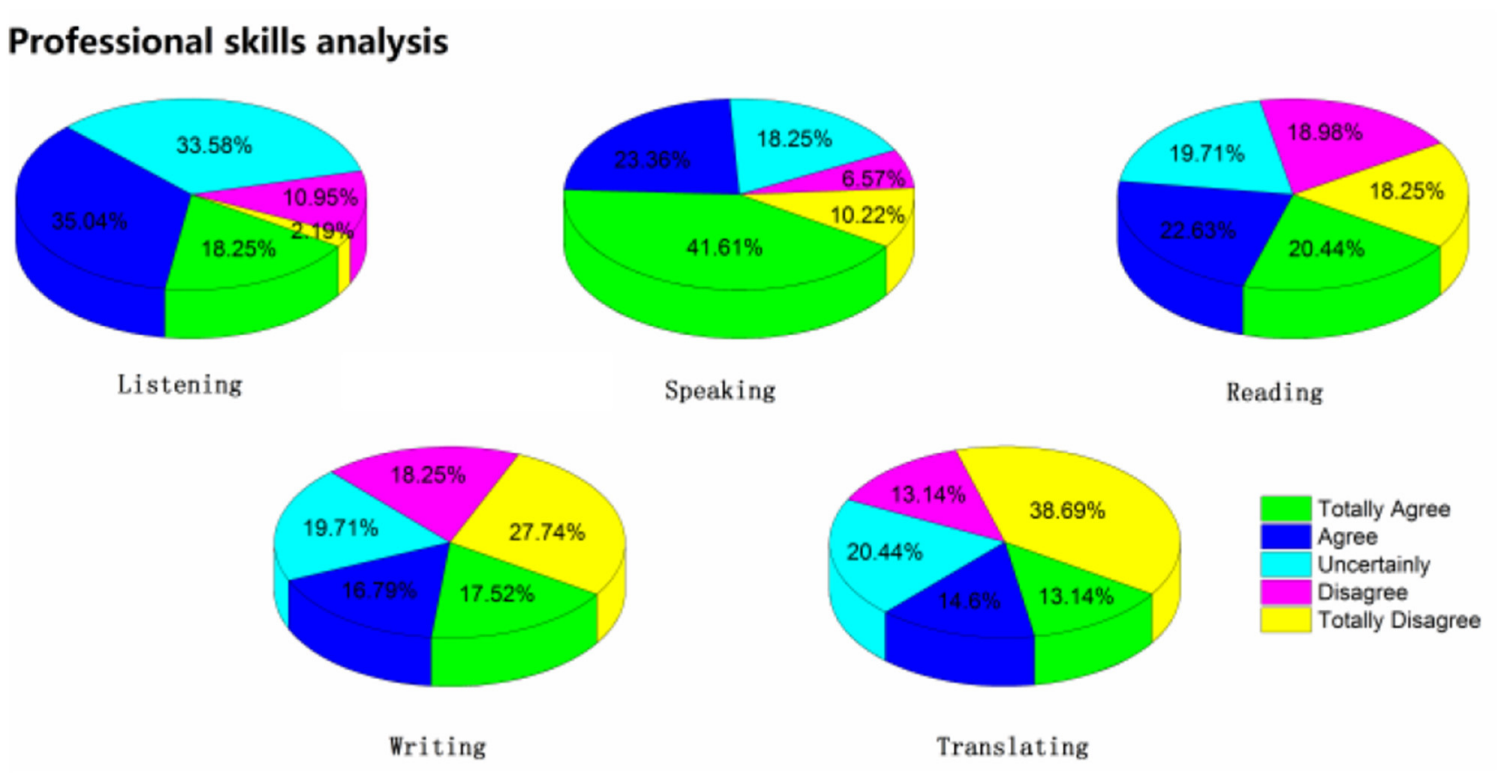
The statistics of students' anxiety in language skills.

From the results of the investigation and analysis, shown in Table 2 and illustrated in Figure 3, 4 in detail. Students' listening and speaking anxiety is significantly higher in the process of online English learning. It coincides with the view of Horwitz and her colleagues that communication ability (speaking and listening) is the main battlefield of foreign language anxiety. It can be seen from the statistical results that the proportion of students' high anxiety is more than 50%, especially in speaking. The proportion of students with high anxiety is 64.97%. Although “speaking” is a great difficulty for students to learn a foreign language, the anxiety of “saying” will be greatly reduced with kindly feedback from teachers and classmates' encouragement in the normal face-to-face classroom. With warm atmosphere, the anxiety sometimes will be temporarily resolved by other emotions. However, in the online course, there is no atmosphere around. Although teachers also encourage students continuously on the other side of the Internet cable, this atmosphere is not really felt. In the independent learning environment, the anxiety of “saying” will be magnified infinitely,” and the anxiety of “listening” is also magnified in the online class. In the process of online class, every listening exercise will make students have a sense of urgency, which cannot be resolved in their own learning environment, so the proportion of high anxiety in this part is as high as 53.29%. The third place is students' reading anxiety, although the rate of high anxiety accounts for 43.07%, not more than half, and the average anxiety is 3.08, which is enough to reflect students' maladjustment to “reading” in online courses. In normal teaching, reading is often the most adaptive and the least anxious part of the students, and reading, to a certain extent, can alleviate the anxiety of learners for its certain recoil reaction time. However, this part may be exaggerated and cannot be ignored in the online class, which shows that the students are more adaptable to the traditional paper-based reading mode, and there are still problems in the adaptability of electronic reading. Compared with the above-mentioned three items, the anxiety of writing and translating, which are generally difficult for students in normal teaching, is not high, and the average anxiety is lower than 3. The survey found that, because these two skills are non-instant skills, there is plenty of time to think about what you do not know, and students will have a certain buffer time to think when completing these two tasks, and this buffer time can greatly alleviate the anxiety of students.

**Table 2:** The statistics of students' anxiety in language skills.

***N***	**Type**	**Totally agree**	**Agree**	**Uncertain**	**Disagree**	**Totally disagree**	**Average**
		**(%)**	**(%)**	**(%)**	**(%)**	**(%)**	
1	Listening	18.25	35.04	33.58	10.95	2.19	3.562
2	Speaking	41.61	23.36	18.25	6.57	10.22	3.796
3	Reading	20.44	22.63	19.71	18.98	18.25	3.080
4	Writing	17.52	16.79	19.71	18.25	27.74	2.781
5	Translating	13.14	14.60	20.44	13.14	38.69	2.504

**Figure 4:**
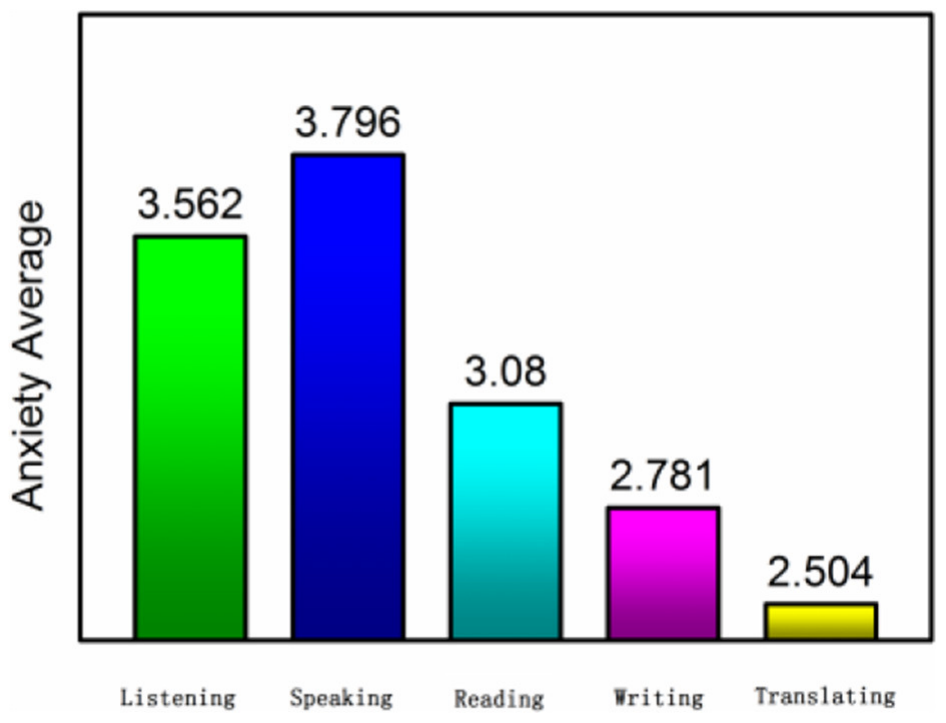
The average value of students' anxiety in language skills.

## Discussion

### Reasons for Students' Psychological Anxiety About Online Courses

Studies have shown that there are various factors causing foreign language anxiety. Horwitz et al. (1986) thought that test anxiety, communication anxiety, and negative evaluation anxiety are the three main sources of anxiety. MacIntyre and Gardner (1991) thought that the main sources of foreign language anxiety are inappropriate teaching mode, fear of negative evaluation, teachers' teaching view, and gender and age. Young (1991) summarized foreign language learning anxiety into six aspects: the relationship between learners and others, learners' beliefs in language learning, teachers' beliefs in language teaching, teacher–student interaction, classroom activity, and testing. Oxford (1999) concluded that the related factors leading to anxiety include self-esteem, concept, classroom activities and ways, and teacher–student interaction. For example, learners who underestimate their foreign language ability are prone to anxiety. Yan and Horwitz (2008) investigated 21 Chinese English-as-a-foreign-language learners and identified seven major causes of foreign language anxiety as regional differences, classroom arrangements, teacher characteristics, learning strategies, test types, parental influence, and peer comparison. Based on the questionnaire and interviews between teachers and students, this study finds that students' online foreign language learning anxiety mainly comes from the following three aspects.

First is learners' motivation. Philips, Liu, and Huang all believe that anxiety has a negative impact on students' attitude and motivation of language learning. The high-anxious students tend to have the lowest expectations of their ability to learn a foreign language (Onwuegbuzie et al., 1999). The higher the level of anxiety, the weaker the motivation of language learning. It is found that students' interest in learning and learning opportunities affect their foreign language learning anxiety to a certain extent (Ganschow and Sparks, 1996; Han, 2018). In the normal face-to-face teaching, students can form a positive learning atmosphere with their learning partners and teachers. Students' interest in learning will be stimulated at this atmosphere, which will enhance the learning motivation. Online learning lacks external motivation, which requires students' internal motivation so much, and the lack of students' internal motivation often leads to the enhancement of psychological anxiety.

The second is learners' learning ability. Foreign language learning ability is a potential factor leading to foreign language learning anxiety. Ganschow et al. found that students with high anxiety think that their language courses are very difficult, while students with low anxiety think that their language courses are very easy. For foreign language learning, the learning circle in face-to-face classroom will make the students with strong learning ability play better and make the students with weak learning ability find some self-confidence driven by the atmosphere so as to be used for expression, communication, and display. However, under the environment of online course, the requirements for learners' individual learning ability are significantly improved; especially for some students with insufficient learning ability, learning will be more difficult, and the anxiety will be greatly enhanced (Ganschow and Sparks, 1996).

The third is the lack of connection between network environment teaching and traditional classroom teaching. It is not the main problem that learners use the Internet to acquire knowledge. The main problems exist in students' expression and communication, the expansion of learners' knowledge, and the test of knowledge points. These problems are actually encountered in actual online teaching, which leads to anxiety. Anxiety leads to tension and fear that waste energy and attention, affect cognition, and then lead to anxiety, which leads to a vicious circle in language learning (Arnold and Brown, 1999), so learners' anxiety is relatively high, and it is necessary to solve the problems in the course in order to reduce it.

### Solutions to Reducing Students' Psychological Anxiety in Online Course

Compared with traditional classroom teaching, the teaching mode under the network environment has higher requirements for learners' autonomous learning ability. According to the analysis of the causes of students' psychological anxiety in the network course and the problems encountered in actual network teaching, the appropriate solutions are conceived. It can be summarized as follows: expanding learning resources (audio, video, and animation, except courseware), organizing group learning (group discussion, group activities, etc.), improving teachers' information quality, enriching extracurricular activities (speech, dubbing, debate, and oral stories), tracking tests (timely testing, post-test analysis, and finding and filling in gaps), and improving learners' network skills (network skills training and the ability to obtain resource expansion).

First of all, one of the reasons for the anxiety of online foreign language courses is the lack of a learning atmosphere, which is the unique advantage of an English normal classroom. Therefore, the establishment of a group learning mode is an excellent choice to create a good mutual learning atmosphere. In the mutual group learning established by the learning office, students can find a familiar and warm atmosphere. Facing the mutual encouragement and encouragement of their partners, students' foreign language learning anxiety will be greatly reduced. Therefore, this paper holds that group learning model is one of the effective strategies to alleviate students' anxiety in foreign language online learning.

Secondly, foreign language learning not only depends on the learning in class but also depends on the acquisition after class. In the normal teaching practice, teachers and students often organize some extra-curricular English activities, such as English corner, imitation show, debate contest, *etc*. However, the organization of online classroom in extracurricular activities is relatively few so that students are exposed to the knowledge of textbooks every day, and students will feel bored for a long time, so an appropriate enrichment of online extracurricular activities is one of the effective ways to reduce students' learning anxiety. Students will find the fun of English in rich activities and find their interest of learning English. The pleasant body and mind with a positive attitude will be effective to reduce anxiety.

Thirdly, richness in learning resources is the incomparable advantage of an Internet course compared with face-to-face learning. Foreign language needs a large amount of practice, and now the developed network can provide all kinds of rich audio and visual reading resources, but in the busy online learning, this advantage is ignored, so teachers can give students proper guidance and encourage students to establish resource sharing, resource mutual assistance, and other forms to make full use of the massive and high-quality network resources. Enriching the network learning resources is essential.

Fourthly, online learning is likely to focus on learning forward without tracking and monitoring the learning effect timely. Therefore, students do not know the degree of their knowledge in consciousness and cannot find a sense of achievement. Therefore, while the online course is being carried out, teachers can consciously enhance the online test links, which can be in various forms, including simple problem tests and complex unit tests. Students can have an overall grasp of the acquired knowledge knowing what they have harvested, which is easy to build self-confidence and obtain a sense of achievement, and learning anxiety will be naturally reduced, so tracking and monitoring are necessary.

Fifthly, one of the problems that cannot be ignored in network learning is the improvement of teachers' network teaching quality and the students' network skills. At present, although the network is no longer a new thing and everyone can operate it freely, most students are more suitable for easy operation. There are many practical functions that cannot be used or cannot be used well when using the network course for learning, resulting in a single form of network teaching and little interest in the network course. Especially the foreign language course needs a lot of interactive activities immediately, such that some teachers and students even have anxiety about the technical problems of online courses. Therefore, improving the network quality of teachers and students is also a good way to reduce learning anxiety.

### The Optimization of the Solution Based on Data Mining

**Figure 5:**
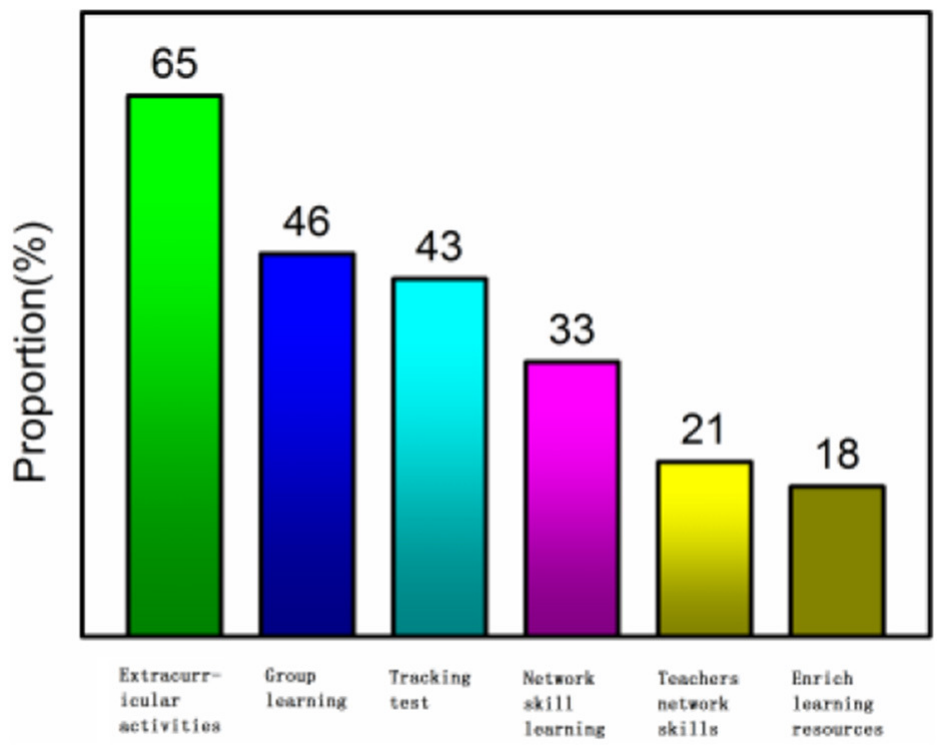
Statistics of methods.

In order to optimize the solution of foreign language learning anxiety on the Internet, the questionnaire is used to count the needs of taking measures and select one or more items by check. The results shown as extracurricular activities (65%), group learning (46%), tracking tests (43%), network skills learning (33%), teachers' network quality (21%), and learning resources (18%) were in an order from high to low as shown in Figure 5.

According to the 160 valid questionnaires collected, we sampled and analyzed the selected students, filtered out the combinations with the same answers, and got a total of 15 groups of different methods combinations. Through sampling and students' comprehensive quality, the heuristic reduction algorithm is used to mine and analyze the data as shown in Table 3. This algorithm belongs to the classic data mining algorithm, which is simple and intuitive. Because the core is the intersection of all the reduction of information system or decision table, this algorithm uses the core as the starting point to calculate the best or user-specified minimum reduction. In the algorithm, the importance of attributes is regarded as a heuristic rule, and the most important attributes are selected to join the kernel according to a certain measure of the importance of attributes. Firstly, attributes are added one by one according to the importance of attributes until the set is a reduction, and then each attribute in the set is checked to see if removing the attribute will change the dependency of the set on the decision attribute; if not, it was deleted. On the premise of keeping the basic problem solving unchanged, the decision-making of curriculum problem is derived through the simplification of curriculum demand design so as to better serve the curriculum design.

**Table 3:** Decision data table of solutions optimization.

**Sample**	**Group learning**	**Enriching learning**	**Extracurricular**	**Teachers' network**	**Network skill**	**Tracking test**	**Decision (d)**
	**(a1)**	**resources (a2)**	**activities (a3)**	**skills (a4)**	**learning (a5)**	**(a6)**	
1	y	y	Y	y	y	y	Y
2	y	y	Y	y	n	n	Y
3	n	y	N	y	n	y	N
4	y	n	Y	n	n	y	Y
5	y	n	Y	y	n	y	Y
6	y	y	Y	n	n	n	Y
7	n	y	N	n	y	n	N
8	y	n	N	y	y	y	Y
9	y	n	Y	y	n	n	Y
10	y	y	N	n	n	y	Y
11	n	n	Y	n	n	y	Y
12	y	y	Y	y	y	n	Y
13	y	y	N	n	y	y	N
14	y	n	Y	n	n	y	Y
15	y	y	N	n	y	n	Y

First, the basic algorithm constructs the discernibility matrix and obtains the discernibility function based on the discernibility matrix. Then, the absorption law is used to simplify the discernibility function and make it a disjunctive normal form, so every main implication is reduction. The basic algorithm can find all the reductions (Mi et al., 2004).

For a decision table, IS = (U, V, f, A∪ {d}), if a property *a* is removed, its positive field changes, that is, POS_(A−{*a*})_(*d*) ≠ POS_*A*_(*d*), where description attribute *a* is the core attribute. Because the *a* relative positive domain of decision attribute *d* is the set of all objects in U that can be accurately divided into {*d*} equivalent classes according to the information of classified U/A, when a certain attribute *a* is removed, the *A*-{*a*} of decision attribute *d* changes with respect to the positive domain, which means that *a* is necessary in *A*, that is, attribute *a* is a core attribute. Conversely, the property *a* is not a core property.

The core of the attribute is found by calculating the relative positive field:

POS_(A−{*a1*})_(*d*) ≠ POS_*A*_(*d*),

POS_(A−{*a2*})_(*d*) = POS_*A*_(*d*),

POS_(A−{*a3*})_(*d*) ≠ POS_*A*_(*d*),

POS_(A−{*a4*})_(*d*) = POS_A_(*d*),

POS_(A−{*a5*})_(*d*) = POS_A_(*d*),

POS_(A−{*a6*})_(*d*) = POS_A_(*d*),

R={*a1*,*a3*}, that is, {Group learning,Extracurricular activities} is the core of data.

U/R = {{1,2,4,5,6,12,14},{3,7},{8,10,15},{9,11},{13}}

U/R∪{*a*2}={{1,2},{3},{4,5},{6,7},{8},{9},{10},{11},{12},{13},{14},{15}}

U/{*d*} = {Y1,Y2},Y1 = {3,7,13},Y2 = {1,2,4,5,6,8,9,10,11,12,14,15}

K_R_(*d*) = 3/15,K_R∪{a2}_(*d*) = 12/15,SIG(*a2*,R,*d*) = 9/15.

U/R∪{*a4*} = {{1,2},{3},{4},{5},{6},{7},{8,9},{10,11},{12},{13},{14,15}}

U/{*d*} = {Y1,Y2},Y1 = {3,7,13},Y2 = {1,2,4,5,6,8,9,10,11,12,14,15}

K_R_(*d*) = 3/15,K_R∪{a4}_(*d*) = 11/15,SIG(*a4*,R,*d*) = 8/15.

U/R∪{*a5*} = {{1},{2},{3},{4},{5,6},{7},{8},{9,10,11},{12},{13},{14},{15}}

U/{*d*} = {Y1,Y2},Y1 = {3,7,13},Y2 = {1,2,4,5,6,8,9,10,11,12,14,15}

K_R_(*d*) = 3/15,K_R∪{a5}_(*d*) = 12/15,SIG(*a5*,R,*d*) = 9/15.

U/R∪{*a6*} = {{1},{2},{3},{4,5},{6},{7},{8,10},{9},{11},{12},{13},{14},{15}}

U/{*d*} = {Y1,Y2},Y1={3,14},Y2={1,2,4,5,6,8,9,10,11,12,14,15}

K_R_(*d*) = 3/15,K_R∪{a6}_(*d*) = 13/15,SIG(*a6*,R,*d*) = 10/15.

For (*a2,a4,a5,a6*), *a2* is 9/15, *a4* is 8/15, *a5* is 9/15, and *a6* is 10/15, among which the *a6* value is the largest. Therefore, *a6* is the most important in these attributes. Thus, add *a6* to subtractive union{A1, A3, A6}. If the threshold of dependency is more than 0.7, the conclusion of termination meets the requirement of subtraction and calculation. The way of extracurricular activities, the mode of group learning, and the tracking test are the core of data, which are the key elements of the six elements. The results calculated by data mining are consistent with the results of the statistical questionnaire analysis. Therefore, the way of extracurricular activities, the mode of group learning, and the tracking test can be regarded as the first solution to solve the students' anxiety.

## Conclusion

Foreign language learning has always been a challenge for many students. Some students always have psychological anxiety about learning a foreign language. In the traditional face-to-face classroom, this anxiety can be more or less relieved by teachers, peers, and various environments. However, in the current era of national online class, students' anxiety about foreign language learning is greatly increased due to the lack of learning ability and learning motivation and the shortage of connection between online courses and traditional courses. Based on the questionnaire of students' psychological anxiety for foreign language learning in the online course, this paper analyzes and compares the current situation of students' anxiety from the aspects of network learning, communication, negative evaluation, and examination and network skills with students' anxiety from the aspects of listening, speaking, reading, writing, and translating and especially finds out that the high rate of communication anxiety coincides with the students' listening and speaking anxiety. Based on the detailed analysis of the reasons for students' anxiety and the results of interview, this paper puts forward six basic countermeasures and issues these six items as a separate questionnaire, making a deep analysis of the survey results with the method of data mining. It was found that the way of extracurricular activities, the model of group learning, and the tracking test are the key factors to effectively reduce students' psychological anxiety in online foreign language learning. It is suggested that teachers can enhance the timeliness and diversity of tests, increase the richness of extracurricular activities, and increase the quality of online teaching and learning of teachers and students to reduce the anxiety of students in an online classroom environment.

## Data Availability Statement

The original contributions presented in the study are included in the article/supplementary material, further inquiries can be directed to the corresponding author.

## Ethics Statement

Ethical review and approval was not required for the study on human participants in accordance with the local legislation and institutional requirements. Written informed consent for participation was not required for this study in accordance with the national legislation and the institutional requirements.

## Author Contributions

XW designed the framework of this research. WZ analyzed the data. Both authors conducted investigation, wrote the manuscript, contributed to the article, and approved the submitted version.

## Conflict of Interest

The authors declare that the research was conducted in the absence of any commercial or financial relationships that could be construed as a potential conflict of interest.
